# Patients undergoing emergent surgery for type A intramural hematomas or type A aortic dissections have similar outcomes

**DOI:** 10.1186/s13019-024-03101-2

**Published:** 2024-10-03

**Authors:** Sorasicha Nithikasem, Abhishek Chakraborty, Hirohisa Ikegami, Manabu Takebe, Gengo Sunagawa, Antonio Chiricolo, Ashok Chaudhary, Alexander Rahimi, Simran Agarwala, Mark Russo, Leonard Y. Lee, Anthony Lemaire

**Affiliations:** Division of Cardiothoracic Surgery, Department of Surgery, RUTGERS-Robert Wood Johnson Medical School, 125 Paterson Street, New Brunswick, NJ 08903 USA

## Abstract

**Objective:**

Despite key differences in pathological processes, both Intramural Hematomas and Aortic Dissections are Acute Aortic Syndromes repaired with similar surgical technique. The objective of this study was to determine differences in surgical outcomes between patients with Intramural Hematoma versus Type A Aortic Dissection undergoing Ascending Aortic Arch repair.

**Methods:**

This retrospective review of prospectively collected data included all patients with acute Intramural Hematoma or Type A Aortic Dissection who underwent emergent Ascending- or Arch Repair from January 2018 to May 2023 at a single academic institution. Primary outcomes included intraoperative mortality, 30-Day mortality, and postoperative stay. Secondary outcomes included postoperative complications. Outcomes were analyzed using Chi-squared, Fisher’s Exact, and t-tests, with significance set at p < 0.05.

**Results:**

A total of 107 patients were included, 27 of whom (25%) had Intramural Hematoma and 80 (75%) had Type A Aortic Dissection. There were no differences in preoperative characteristics such as age, gender, and comorbidities, and no differences in perioperative characteristics such as case length, cardiopulmonary bypass, aortic cross-clamp, and circulatory arrest times. When comparing postoperative outcomes, there was a higher rate of postoperative pericardial effusions requiring pericardial window in the Intramural Hematoma cohort compared to the Aortic Dissection cohort (15% [n = 4] vs. 3% [n = 2]; p = 0.02). There were no differences in other primary outcomes such as intraoperative mortality, 30-Day mortality, and postoperative length of stay. There were also no differences in the rates of postoperative complications such as bleeding requiring reoperation, cerebrovascular accident, atrial fibrillation, pleural effusion requiring thoracentesis, and surgery-related Emergency Department visits.

**Conclusions:**

Our analysis demonstrates similar outcomes for patients undergoing Ascending Aortic Arch repair between patients with Intramural Hematoma and Type A Aortic Dissection. Despite the higher rate of required postoperative pericardial windows in the Intramural Hematoma cohort, the overall primary outcomes remained comparable. These findings better elucidate the standard of care for patients with acute Intramural Hematoma undergoing Ascending Aortic Arch repair.

## Introduction

Acute aortic syndromes (AAS) include life-threatening pathologies such as Aortic Dissection (AD) and Intramural Hematoma (IMH). IMH comprises 10–30% of AAS and its progression and surgical management is controversial [1]. Although the pathology of IMH has been well documented there are a significant percentage of medical professionals that do not have a great understanding of the etiology. It is suggested that aortic IMH is hemorrhage within the aortic wall as result of ruptured vaso-vasorum. Another theory states that IMH is caused by intimal tears that are too small to detect on a standard CTA-scan, with a thrombosed false lumen [1]. IMH has traditionally been challenging to diagnose and understand. The distinction between an aortic dissection and an IMH has been very difficult for not only medical professionals but also surgeons. Currently, patients with IMH are treated like patients with an aortic dissection. The patients with IMH of the ascending aorta are generally treated with surgical intervention. In contrast, patients with IMH of the descending aorta are treated with medical management unless they meet specific criteria for intervention. The surgical outcomes of patients with IMH compared to patients with aortic dissections are not clear. The purpose of the study is to determine if there are differences in surgical outcomes between patients with an IMH and aortic dissections.

## Methods

This was a retrospective review that included patients with acute IMH or Type A Aortic Dissection who underwent emergent Ascending Aortic and Arch Repair from January 2018 to May 2023. The patients with IMH were classified as type A IMH as all the patients had involvement of the ascending aorta with IMH. The study was performed at a single academic institution. The surgeries were all performed within 1–2 h of diagnosis. Primary outcomes included intraoperative mortality, 30-Day mortality, and postoperative stay. Secondary outcomes included postoperative complications. Outcomes were analyzed using Chi-squared, Fisher’s Exact, and t-tests, with significance set at p < 0.05.

## Results

A total of 107 patients were included, 27 of whom (25%) had Intramural Hematoma and 80 (75%) had Type A Aortic Dissection. The results of the study showed that there were no differences in characteristics such as age, gender, race, and Body Mass Index (Table 1). In addition, no differences in preoperative comorbidities such as hypertension, dyslipidemia, diabetes mellitus Type 2, smoking history, and drug abuse history. There were no differences in other primary outcomes such as intraoperative mortality, and 30-Day mortality. Furthermore, postoperative length of stay, or in perioperative characteristics such as case length, bypass time, circulatory arrest time, and cross-clamp time were equivalent (Table 2). Interestingly, there was a higher rate of pericardial effusions requiring pericardial window in the Intramural Hematoma cohort compared to the Aortic Dissection cohort (15% [n = 4] vs. 3% [n = 2]; p = 0.02. The requirement of additional surgery had no impact on mortality. Moreover, there were no differences in postoperative complications such as bleeding requiring reoperation, cerebrovascular accident, atrial fibrillation, pleural effusion requiring thoracentesis, and surgery-related Emergency Department visits. The surgery performed in each patient was an ascending aortic and Hemi-arch repair.

**Table 1:** Baseline characteristics

Variable	Overall	Intramural Hematoma	Aortic Dissection	P-value
	**(n = 107)**	**(n = 27)**	(n = **80)**	
*Baseline characteristics*				
Age (years) (Median, IQR)	63 (54—74)	64 (58—75)	63(53—73)	0.184
Gender (male) n (%)	44 (74%)	15 (75%)	29 (74%)	0.782
Race (White Non-Hispanic) n (%)	66 (62%)	19(70%)	47 (59%)	0.283
Body Mass index (Median, IQR)	27 (24—32)	27 (22—32)	28 (24—32)	0.162
*Comorbidities*				
Hypertension n (%)	99 (93%)	27(100%)	72 (90%)	0.09
Dyslipidemia n (%)	46 (43%)	9 (33%)	37 (46%)	0.241
Diabetes Mellitus Type I/II n (%)	8 (7%)	2 (7%)	6 (8%)	0.987
Smoking History n (%)	41 (38%)	10 (37%)	31 (39%)	0.874
Drug Abuse History n (%)	6 (6%)	2 (7%)	4 (8%)	0.641

Indicates significance at p < 0.05

**Table 2:** Outcomes and complications

Variable	Overall	(Intramural Hematoma	Aortic Dissection	P-value
	**(n = 107)**	**(n = 27)**	**(n = 80)**	
*Outcomes*				
Intraoperative Mortality n (%)	6 (6%)	0 (0%)	6 (8%)	0.334
30-Day Mortality n (%)	21 (19%)	3 (11%)	18 (23%)	0.198
Postoperative Length of Stay n (%)	9 (5–15)	10 (7—18)	8 (5–13)	0.08
*Perioperative characteristics*				
Case Length (minutes)	278 (238—356)	278 (243—346)	278 (231–362)	0.420
Cardiopulmonary Bypass Time (minutes)	143 (122–190)	144 (122—202)	143 (120–185)	0 449
Circulatory Arrest Time (minutes)	22 (18–28)	24 (17–30)	22 (18–27)	0.336
Aortic Cross-clamp Time (minutes)	92 (75- 125)	92 (75—147)	92 (75–125)	0.426
*Postoperative complications*				
Postoperative Bleeding Requiring Intervention n (%)	30 (28%)	7 (26%)	23 (29%)	0.489
Postoperative Cerebrovascular Accident n (%)	21 (20%)	8 (30%)	13 (16%)	0.231
Postoperative Atrial Fibrillation n (%)	27 (25%)	7 (26%)	20 (25%)	0.7X2
Postoperative Pericardial Window n (%)	6 (6%)	4 (15%)	2 (3%)	0.020*
Postoperative Thoracentesis n (%)	25 (23%)	7 (26%)	18 (23%)	0.990
Surgery-Related Emergency Department Visit n (%)	43 (40%)	11 (41%)	32 (40%)	0.631

Indicates significance at p < 0.05

## Conclusions

In this limited cohort, there were no significant differences in the surgical outcomes of patients with an acute type A AD or type A IMH that underwent emergent surgical repair. This study compared only those type A IMH patients that underwent surgery rather than conservative management. These results may be considered surprising for medical professionals that know that there is a difference in pathology. The patients are technically treated the same way however the intraoperative findings are vastly different. All patients with aortic dissections are technically identified as having an intimal flap. The location of the flap and the ability to resect it can impact the complexity of the case. For example, the patients with ADs that have intimal flaps that extend into the aortic root, can sometimes require an aortic root reconstruction. The lack of an intimal tear often decreases the need for additional complex surgery. Furthermore, the patients with ADs often have pericardial effusions that can lead to cardiac tamponade and ultimate death.

As expected, a smaller portion of the study population had IMH with only 25% (N = 27) of the patients in the cohort compared to patients with ascending aortic dissections. The lower percentage of patients with IMH is a common finding in most medical centers as IMH is not as frequent as AD. The patient population was similar without major differences in co-morbidities or demographic data. Despite the difference in surgical pathology the surgical outcomes are essentially the same. These findings of this study are supported in the literature by Evangelista et al. [2]. In this study, the authors observed similar outcomes for patients undergoing Ascending Aortic Arch repair between patients with Intramural Hematoma and Type A Aortic Dissection. Among the 1,010 patients, 58 (5.7%) met the strict criterion of acute IMH. The patients with IMH tended to be older than those with classic AD (P < 0.001), and the majority (60%) of IMHs were located in the descending aorta. Abrupt onset of severe chest or back pain was the most common presenting symptom for both IMH and AD, and time from symptom onset to presentation was similar for IMH and AD. **A**ortic insufficiency was seen infrequently in IMH, and pulse deficits were also less common than in AD. Compared with patients with AD, patients with IMH were more likely to have a normal ECG (45.6% versus 29.8%; P0.012), and no patient with an acute IMH had an acute myocardial infarction [2]. The overall hospital mortality for IMH was similar to that of AD (20.7% versus 23.9%; P0.57). In particular, IMH of the ascending aorta carried an in-hospital mortality of 39.1%, nonsignificantly higher than that of AD, a condition more frequently treated surgically. Mortality for IMH involving the ascending aorta was 42.9% with surgical therapy and 33.3% with medical management, as opposed to a mortality rate of 24.2% with surgical therapy in AD involving the ascending aorta and 56.5% with medical therapy alone.

Acute aortic IMH is defined by the hemorrhage within the aortic wall without intimal tear, which could develop into an AD and rupture. Retrograde ascending aortic intramural hematoma (RAIMH) is a special type of intramural hematoma that is characterized by the presence of a primary tear or ulcer at the distal end of the aortic arch, with involvement of the ascending aorta and even the aortic arch [3]. Further investigation into this type of IMH is required to see if there are similar findings to antegrade IMH patients. IMH is a similar entity to acute aortic dissection but lacks two criteria seen in acute dissection: an evident intimal tear and a perfused false lumen [4]. IMH is thought to occur either from spontaneous rupture of the vasa vasorum within the aortic media, leading to medial bleeding that propagates in a manner similar to dissection and eventually tamponades, or from an intimal tear too small to be discerned on imaging studies that generates a thrombosed false lumen [5].

Finally, the treatment of patients with IMH has mirrored the treatment of patients with acute aortic dissections. The presence of IMH of the ascending aorta often requires replacement of the ascending aorta and hemiarch repair. Acute aortic syndromes are a highly morbid set of conditions characterized by the sudden onset of thoracic pain and includes aortic dissection and intramural aortic hemorrhage with a variable clinical course [6, 7]. Although there is consensus about the definition and treatment of AD the approach to acute IMH of the aorta remains elusive. Much of the controversy stems from an incomplete knowledge of its natural history [8, 9]. The treatment of patients with IMH and acute aortic dissection have similar outcomes despite the technical differences in the pathology. These findings are important in terms of discussing prognosis and discussions with the patient and family members.

Taken together, the results from the study show that the focus of medical professionals should be to improve surgical outcomes for both groups by decreasing morbidity and mortality rates. Furthermore, more emphasis should be placed on educating medical professionals and patients on the different aortic syndromes as there continues to be confusion on the multiple types.

## Data Availability

No datasets were generated or analysed during the current study.
